# Childhood aggression and the co-occurrence of behavioural and emotional problems: results across ages 3–16 years from multiple raters in six cohorts in the EU-ACTION project

**DOI:** 10.1007/s00787-018-1169-1

**Published:** 2018-05-29

**Authors:** Meike Bartels, Anne Hendriks, Matteo Mauri, Eva Krapohl, Alyce Whipp, Koen Bolhuis, Lucia Colodro Conde, Justin Luningham, Hill Fung Ip, Fiona Hagenbeek, Peter Roetman, Raluca Gatej, Audri Lamers, Michel Nivard, Jenny van Dongen, Yi Lu, Christel Middeldorp, Toos van Beijsterveldt, Robert Vermeiren, Thomas Hankemeijer, Cees Kluft, Sarah Medland, Sebastian Lundström, Richard Rose, Lea Pulkkinen, Eero Vuoksimaa, Tellervo Korhonen, Nicholas G. Martin, Gitta Lubke, Catrin Finkenauer, Vassilios Fanos, Henning Tiemeier, Paul Lichtenstein, Robert Plomin, Jaakko Kaprio, Dorret I. Boomsma

**Affiliations:** 1 Netherlands Twin Register, Department of Biological Psychology, Vrije Universiteit Amsterdam, van der Boechorststraat 1, 1081 BT Amsterdam, The Netherlands; 2 Amsterdam Public Health Research Institute, Faculty of Behavioural and Movement Sciences, Vrije Universiteit Amsterdam, Amsterdam, The Netherlands; 3 Amsterdam Neuroscience, Amsterdam, The Netherlands; 4 University of Cagliari, Cagliari, Italy; 5 Medical Research Council Social, Genetic, and Developmental Psychiatry Centre, Institute of Psychiatry, Psychology, and Neuroscience, King’s College London, London, UK; 6 Institute for Molecular Medicine Finland, University of Helsinki, Helsinki, Finland; 7 Department of Epidemiology, Erasmus Medical Center, Rotterdam, The Netherlands; 8 Psychiatric Genetics Laboratory, QIMR Berghofer Medical Research Institute, Brisbane, Australia; 9 Department of Psychology, University of Notre Dame, Notre Dame, USA; 10 Curium-LUMC, Academic Centre of Child and Youth Psychiatry, Leiden University Medical Center, Leiden, The Netherlands; 11 Department of Medical Epidemiology and Biostatistics, Karolinska Institutet, Stockholm, Sweden; 12 University of Queensland, Brisbane, Australia; 13 VU Medical Centre, Amsterdam, The Netherlands; 14 Division of Analytical Biosciences, Leiden Academic Centre for Drug Research, Leiden University, Leiden, The Netherlands; 15 Good Biomarker Sciences, Leiden, The Netherlands; 16 Gillberg Neuropsychiatry Centre, University of Gothenburg, Gothenburg, Sweden; 17 Centre for Ethics, Law and Mental Health (CELAM), University of Gothenburg, Gothenburg, Sweden; 18 Department of Psychological & Brain Sciences, Indiana University, Bloomington, IN USA; 19 Department of Psychology, University of Jyvaskyla, Jyvaskyla, Finland; 20 Department of Public Health, University of Helsinki, Helsinki, Finland; 21 Institute of Public Health and Clinical Nutrition, University of Eastern Finland, Kuopio, Finland; 22 QIMR Berghofer Medical Research Institute, Brisbane, QLD Australia; 23 Youth Studies, Interdisciplinary Center, Utrecht University, Utrecht, The Netherlands; 24 Department of Child and Adolescent Psychiatry, Erasmus Medical Center, Rotterdam, The Netherlands; 25 Department of Psychiatry, Erasmus Medical Center, Rotterdam, The Netherlands

**Keywords:** Aggression, Childhood, Comorbidity, Co-occurence, Behavioural and emotional problems

## Abstract

Childhood aggression and its resulting consequences inflict a huge burden on affected children, their relatives, teachers, peers and society as a whole. Aggression during childhood rarely occurs in isolation and is correlated with other symptoms of childhood psychopathology. In this paper, we aim to describe and improve the understanding of the co-occurrence of aggression with other forms of childhood psychopathology. We focus on the co-occurrence of aggression and other childhood behavioural and emotional problems, including other externalising problems, attention problems and anxiety–depression. The data were brought together within the EU-ACTION (Aggression in Children: unravelling gene-environment interplay to inform Treatment and InterventiON strategies) project. We analysed the co-occurrence of aggression and other childhood behavioural and emotional problems as a function of the child’s age (ages 3 through 16 years), gender, the person rating the behaviour (father, mother or self) and assessment instrument. The data came from six large population-based European cohort studies from the Netherlands (2x), the UK, Finland and Sweden (2x). Multiple assessment instruments, including the Child Behaviour Checklist (CBCL), the Strengths and Difficulties Questionnaire (SDQ) and Multidimensional Peer Nomination Inventory (MPNI), were used. There was a good representation of boys and girls in each age category, with data for 30,523 3- to 4-year-olds (49.5% boys), 20,958 5- to 6-year-olds (49.6% boys), 18,291 7- to 8-year-olds (49.0% boys), 27,218 9- to 10-year-olds (49.4% boys), 18,543 12- to 13-year-olds (48.9% boys) and 10,088 15- to 16-year-olds (46.6% boys). We replicated the well-established gender differences in average aggression scores at most ages for parental ratings. The gender differences decreased with age and were not present for self-reports. Aggression co-occurred with the majority of other behavioural and social problems, from both externalising and internalising domains. At each age, the co-occurrence was particularly prevalent for aggression and oppositional and ADHD-related problems, with correlations of around 0.5 in general. Aggression also showed substantial associations with anxiety–depression and other internalizing symptoms (correlations around 0.4). Co-occurrence for self-reported problems was somewhat higher than for parental reports, but we found neither rater differences, nor differences across assessment instruments in co-occurrence patterns. There were large similarities in co-occurrence patterns across the different European countries. Finally, co-occurrence was generally stable across age and sex, and if any change was observed, it indicated stronger correlations when children grew older. We present an online tool to visualise these associations as a function of rater, gender, instrument and cohort. In addition, we present a description of the full EU-ACTION projects, its first results and the future perspectives.

## Introduction

Prevention strategies and behavioural and pharmacological interventions for aggressive behaviour and conduct disorder are effective in some children, although a substantial number of children do not respond to prevention strategies, do not benefit from interventions or may even experience an escalation of symptom [9, 10]. One reason for this might be the heterogeneity of aggression. A second reason, which is related to the heterogeneous nature and occurrence of childhood aggressive problems, might be that children with aggressive problems often have co-occurring problems. Due to a multitude of problems, children may not respond to prevention or intervention targeting aggression, or the co-occurring problems may mask aggression, leaving it untreated. In 12 year olds, Bartels and colleagues [11] observed that at least half of the children who were deviant on aggressive behaviour (T score ≥ 67) also were deviant on rule-breaking behaviour, i.e. at least 50% of the children with clinical levels of aggression also showed a co-occurrence of clinically relevant rule-breaking behaviour. Strong links between aggression and attention-deficit/hyperactivity disorder (ADHD) [12] are often seen in the clinical presentation of ADHD [13], and it has been suggested that the strong association between ADHD and aggression may explain gender differences in clinical referral. For example, teachers rated boys with a DSM-based ADHD diagnosis as having higher levels of attention problems and aggression than girls with a similar ADHD diagnosis [14]. Aggression not only co-occurs with psychopathologies on the externalizing spectrum. Aggression also tends to co-occur with anxiety, and it has been proposed that anxiety needs to be given a central role in the treatment of aggression [15]. In more extreme cases, aggression was not found to co-occur solely with ADHD symptoms, such as attention problems, or anxiety but rather with both of these forms of psychopathology. This pattern of behavioural problems is referred to as the dysregulation profile [16–18], and has been described as a potential marker for severe childhood psychopathologies [19, 20].

To gain insight into the aetiology of individual differences in childhood aggression and in co-occurring behavioural and emotional problems, ACTION (Aggression in Children: unravelling gene-environment interplay to inform Treatment and InterventiON strategies; http://www.action-euproject.eu/) created a consortium with access to large childhood prospective twin, population-based and clinical cohorts. ACTION brings together multiple large cohort studies in genetically informative populations (see Table 1 and Appendix 1). The focus of ACTION is to inform on the aetiology of differences in aggression between children by unravelling its genetic architecture using univariate, multivariate and longitudinal genetic and epigenetic modelling in twin and genetic and epigenetic association studies. A strong focus of ACTION includes biomarker and metabolomics research [21].

**Table 1:** Sample sizes for different age groups of the ACTION cohort

Register	Age											Webpages
	1–2	3–4	5–6	7–8	9–10	11–12	13–14	15–16	17–18	19–20	21–22	
NTR	106.7	37.9	31.2	23.2	18.1	15.1	8.0	5.7	1.7	6.0		http://www.Tweelingenregister.org
Qtwin						2.4	1.4	1.8			0.9	http://www.qimrberghofer.edu.au/qtwin/
TEDS	12.6	28.4		29.2	6.8	11.8	6.7	10.2				http://www.Teds.ac.uk
TCHAD				2.0			2.0	2.0		2.0		http://ki.se/en/meb/twin-study-of-child-and-adolescent-development-tchad
CATSS					22.3	6.5		11.1	8.7			http://ki.se/en/meb/the-child-and-adolescent-twin-study-in-sweden-catss
FT12						5.3	4.7		4.2		1.3	https://wiki.helsinki.fi/display/twineng/Twinstudy
GenR	4.5	5.2	7.8		5.0							http://www.generationr.nl
Indiv (x 1000)	123.8	71.5	39	54.4	40.4	45	22.8	24.7	8.1	8	2.2	

In the current study, the aim is to describe and improve the understanding of the co-occurrence of aggression with other forms of childhood psychopathology by analysing data from the large ACTION phenotype databases in large samples of children. We analysed data on aggression and common emotional and behavioural problems in children aged 3–16 years. Multiple raters, i.e. fathers and mothers during childhood and also youngsters themselves during adolescence, provided information on different aggression measures. The two Dutch cohorts (The Netherlands Twin Register and Generation R) used the Achenbach System of Empirically Based Assessment (ASEBA [22]), which included the Child Behaviour Checklist (CBCL) and the Youth Self-Report (YSR). The UK-based Twins Early Development Study employed the Strengths and Difficulties Questionnaire (SDQ [23]). The Swedish Twin study of Child and Adolescent Development used the Autism–Tics, ADHD and other Comorbidities inventory (A-TAC [24]), and the Swedish Child and Adolescent Twin Study the ASEBA questionnaires. In Finland, the Multidimensional Peer Nomination Inventory (MPNI) was employed. For several age groups from different countries, aggression assessed with identical instruments was available. For example, parental ratings with the CBCL were available for 7- to 8-year-olds and 12- to 13-year-olds in the Netherlands (NTR) and Sweden (TCHAD). In addition to indicators of aggression, all instruments provided quantitative scores on other childhood psychopathologies from the externalising and internalising spectrum. We investigated patterns of co-occurrence across age, rater, instrument and gender.

## Methods

### Participants

Six large population-based cohorts (NTR and GenR from the Netherlands, TEDS from the UK, CATSS and TCHAD from Sweden and FinnTwin12 from Finland) analysed the co-occurrence of aggression measures with other psychopathologies. For a link to cohort-specific websites, see Table 1 and for a detailed description of the cohorts, please also see Appendix I. The twin cohorts were requested to randomly select one of the twins per pair, with an equal representation of first- and second-born children, to obtain parameter estimates that were not biased due to effects of family clustering. In our previous work [25], we have shown that children with an illness or disability that interfered with daily function tend to display more than twice as much problem behaviour across the entire age range compared to other twins, so they were excluded. Age-, gender- and rater- specific sample sizes are presented in Tables 2, 3, 4 and 5. Data were available for 30,523 3- to 4-year-olds (49.5% boys), 20,958 5- to 6-year-olds (49.6% boys), 18,291 7- to 8-year-olds 49% boys), 27,218 9- to 10-year-olds (49.4% boys), 18,543 11- to 12-year-olds (48.9% boys) and 10,088 15- to 16-year- olds (46.6% boys). Due to the longitudinal structure of most cohorts, these data points are not statistically independent observations, since overlapping groups of children were assessed at multiple ages. All data used in the current analyses were collected under protocols that have been approved by the appropriate ethics committees, and studies were performed in accordance with the ethical standards laid down in the 1964 Declaration of Helsinki and its later amendments.

**Table 2:** Means and standard deviations for the empirical scales of the Child Behaviour Checklist (CBCL) 1.5–5

ASEBA-CBCL 1.5–5	Rater	Age	Sex	N	Aggressive behaviour	Attention problems	Withdrawn	Anxious– depressed	Emotional reactivity	Somatic complaints	Sleep problems
Gen R	Mother	3	Boy	2271	7.58 (5.37)	1.56 (1.64)	0.98 (1.43)	1.08 (1.56)	1.67 (1.82)	1.61 (1.61)	1.98 (2.16)
		3	Girl	2246	6.37 (4.91)	1.44 (1.56)	0.84 (1.24)	0.99 (1.48)	1.55 (1.79)	1.57 (1.74)	1.92 (2.09)
Gen R	Father	3	Boy	1840	8.24 (5.60)	1.80 (1.72)	1.03 (1.40)	1.16 (1.54)	1.86 (1.99)	1.64 (1.79)	2.08 (2.24)
		3	Girl	1897	7.14 (5.03)	1.55 (1.61)	0.93 (1.27)	1.10 (1.52)	1.67 (1.89)	1.52 (1.67)	1.91 (2.04)
NTR	Mother	3	Boy	9277	11.48 (6.85)	2.34 (1.97)	1.47 (1.69)	1.95 (1.99)	2.92 (2.66)	1.76 (1.84)	1.86 (2.18)
		3	Girl	9360	9.95 (6.30)	2.03 (1.84)	1.39 (1.56)	2.05 (2.00)	2.96 (2.57)	1.86 (1.92)	1.93 (2.20)
Gen R	Mother	6	Boy	2887	6.65 (5.79)	1.84 (1.85)	1.33 (1.64)	1.51 (1.93)	1.97 (2.38)	1.56 (1.89)	1.49 (1.93)
		6	Girl	2856	5.93 (4.90)	1.30 (1.59)	1.02 (1.35)	1.46 (1.83)	1.68 (2.06)	1.61 (1.89)	1.51 (1.93)

**Table 3:** Means and standard deviations for the empirical scales of the ASEBA taxonomy (CBCL and YSR)

ASEBA 6–18	Rater	age	Sex	N	Aggressive behaviour	Attention problems	Rule breaking	Social problems	Anxious– depressed	Withdrawn– depressed	Thought problems	Somatic complaints
NTR	Mother	7	Boy	5720	5.74 (5.29)	3.48 (3.13)	1.58 (2.02)	2.17 (2.48)	2.12 (2.53)	1.14 (1.63)	1.66 (2.14)	1.10 (1.57)
		7	Girl	5853	4.38 (4.28)	2.56 (2.79)	1.07 (1.55)	1.91 (2.24)	2.31 (2.58)	1.09 (1.53)	1.29 (1.77)	1.26 (1.68)
NTR	Father	7	Boy	4134	4.98 (4.75)	3.13 (2.97)	1.37 (1.85)	1.80 (2.18)	1.62 (2.04)	0.95 (1.45)	1.33 (1.85)	0.81 (1.28)
		7	Girl	4182	3.81 (4.01)	2.26 (2.59)	0.95 (1.47)	1.58 (1.95)	1.77 (2.19)	0.86 (1.36)	0.91 (1.46)	0.91 (1.39)
TCHAD	Parent	8	Boy	552	5.49 (5.42)	1.91 (2.40)	1.18 (1.63)	0.99 (1.57)	1.74 (2.61)	0.99 (1.24)	0.13 (0.59)	0.56 (1.04)
		8	Girl	534	4.77 (4.89)	1.32 (2.07)	0.79 (1.28)	0.84 (1.52)	2.01 (2.75)	1.13 (1.35)	0.13 (0.53)	0.75 (1.28)
NTR	Mother	9	Boy	4543	5.09 (5.16)	3.43 (3.21)	1.43 (2.06)	2.05 (2.54)	2.14 (2.67)	1.24 (1.75)	1.61 (2.14)	1.07 (1.59)
		9	Girl	4689	3.94 (4.20)	2.42 (2.75)	0.93 (1.55)	1.83 (2.34)	2.39 (2.80)	1.13 (1.64)	1.25 (1.80)	1.28 (1.78)
NTR	Father	9	Boy	3210	4.18 (4.57)	3.07 (3.11)	1.17 (1.78)	1.72 (2.33)	1.65 (2.21)	1.00 (1.60)	1.27 (1.88)	0.83 (1.38)
		9	Girl	3255	3.34 (3.82)	2.17 (2.64)	0.79 (1.38)	1.55 (2.07)	1.86 (2.33)	0.91 (1.46)	0.93 (1.48)	0.88 (1.39)
Gen R	Mother	10	Boy	2250	3.26 (4.08)	3.76 (3.35)	1.24 (1.67)	1.84 (2.35)	2.14 (2.72)	1.29 (1.78)	1.80 (2.36)	1.34 (1.92)
		10	Girl	2310	2.54 (3.34)	2.81 (3.00)	0.81 (1.27)	1.62 (2.13)	2.28 (2.64)	1.01 (1.48)	1.50 (2.01)	1.59 (2.02)
Gen R	Father	10	Boy	1624	3.28 (4.16)	3.81 (3.31)	1.36 (1.69)	1.96 (2.35)	2.05 (2.54)	1.36 (1.74)	1.92 (2.39)	1.25 (1.72)
		10	Girl	1670	1.47 (3.24	2.87 (2.81)	0.89 (1.30)	1.71 (2.01)	2.11 (2.58)	1.00 (1.48)	1.43 (1.82)	1.41 (1.80)
NTR	Mother	12	Boy	3870	4.18 (4.63)	3.21 (3.20)	1.28 (1.87)	1.72 (2.45)	1.90 (2.56)	1.25 (1.81)	1.36 (2.02)	0.89 (1.41)
		12	Girl	4010	3.27 (3.82)	2.12 (2.59)	0.79 (1.37)	1.46 (2.17)	2.18 (2.70)	1.10 (1.76)	1.03 (1.64)	1.03 (1.58)
NTR	Father	12	Boy	2764	3.65 (4.36)	3.02 (3.14)	1.15 (1.78)	1.58 (2.39)	1.59 (2.39)	1.10 (1.76)	1.12 (1.77)	0.72 (1.26)
		12	Girl	2839	2.80 (3.52)	1.95 (2.49)	0.71 (1.29)	1.23 (1.90)	1.70 (2.29)	0.95 (1.58)	0.77 (1.35)	0.73 (1.26)
TCHAD	Parent	13	Boy	535	3.90 (4.30)	1.59 (2.08)	1.14 (1.56)	0.79 (1.29)	1.28 (2.01)	1.01 (1.31)	0.13 (0.60)	0.62 (1.13)
		13	Girl	522	3.71 (4.60)	1.21 (1.97)	0.82 (1.52)	0.75 (1.53)	2.06 (3.31)	1.27 (1.64)	0.18 (0.67)	0.78 (1.37)
TCHAD	Self	13	Boy	560	8.07 (4.96)	3.57 (2.78)	2.94 (2.26)	2.02 (2.05)	3.78 (3.72)	2.12 (1.88)	1.31 (1.53)	1.47 (1.76)
		13	Girl	551	7.94 (4.33)	3.75 (2.64)	2.64 (2.36)	1.89 (1.80)	5.09 (4.75)	2.42 (1.91)	1.76 (1.98)	2.19 (2.36)
TCHAD	Parent	16	Boy	532	3.06 (3.83)	1.24 (1.85)	1.12 (1.54)	0.57 (1.08)	1.14 (1.87)	0.90 (1.27)	0.10 (0.41)	0.65 (1.15)
		16	Girl	507	3.25 (3.97)	1.21 (1.98)	1.11 (1.94)	0.55 (1.17)	2.18 (3.44)	1.11 (1.51)	0.18 (0.68)	1.09 (1.78)
TCHAD	Self	16	Boy	583	7.10 (4.38)	3.44 (2.69)	2.93 (2.23)	1.77 (1.91)	2.97 (3.61)	2.01 (1.89)	1.08 (1.65)	1.21 (1.60)
		16	Girl	606	7.77 (4.41)	4.14 (2.69)	3.02 (2.39)	1.78 (1.76)	5.60 (4.66)	2.85 (2.07)	1.45 (1.78)	2.34 (2.50)

**Table 4:** Means and standard deviations for the scales of the Strengths and Difficulties Questionnaire (SDQ)

SDQ	Rater	Age	Sex	N	Conduct problems	Hyperactivity	Peer problems	Emotion–anxiety	Prosocial
TEDS	Parent	4	Boy	3581	2.23 (1.58)	4.35 (2.34)	1.58 (1.51)	1.35 (1.39)	7.07 (1.85)
		4	Girl	3788	1.93 (1.49)	3.64 (2.20)	1.34 (1.41)	1.42 (1.47)	7.66 (1.77)
TEDS	Parent	7	Boy	2740	1.89 (1.73)	3.94 (2.61)	1.05 (1.46)	2.02 (1.74)	7.93 (1.84)
		7	Girl	2892	1.45 (1.47)	3.09 (2.35)	0.83 (1.23)	2.28 (1.82)	8.54 (1.55)
TEDS	Parent	9	Boy	1055	1.35 (1.43)	3.56 (2.45)	1.05 (1.56)	1.47 (1.67)	7.91 (1.85)
		9	Girl	1245	1.08 (1.30)	2.68 (2.08)	0.91 (1.33)	1.82 (1.88)	8.67 (1.48)
TEDS	Self	9	Boy	1055	2.39 (1.89)	4.13 (2.72)	1.93 (1.74)	2.99 (2.28)	7.39 (1.95)
		9	Girl	1245	1.92 (1.69)	3.43 (2.15)	1.76 (1.71)	3.38 (2.40)	8.38 (1.62)
TEDS	Parent	12	Boy	1828	1.42 (1.48)	3.33 (2.36)	1.18 (1.58)	1.67 (1.80)	8.25 (1.74)
		12	Girl	2117	1.16 (1.33)	2.28 (1.99)	0.93 (1.35)	1.90 (1.94)	8.86 (1.50)
TEDS	Self	12	Boy	1828	2.09 (1.48)	3.85 (2.33)	1.47 (1.63)	1.94 (1.93)	6.98 (1.96)
		12	Girls	2117	1.64 (1.50)	3.09 (2.16)	1.22 (1.48)	2.43 (2.10)	7.95 (1.69)
CATSS	Parent	15	Boys	2083	0.93 (1.21)	2.34 (2.23)	1.29 (1.66)	0.83 (1.34)	8.03 (1.85)
		15	Girls	2199	0.99 (1.30)	1.72 (1.93)	1.21 (1.61)	1.43 (1.76)	8.49 (1.80)
CATSS	Self	15	Boys	2258	1.78 (1.52)	3.42 (2.19)	1.79 (1.55)	2.00 (1.80)	7.37 (1.88)
		15	Girls	2806	1.73 (1.39)	3.42 (2.19)	1.79 (1.55)	2.00 (1.80)	7.37 (1.88)
TEDS	Parent	16	Boys	2134	1.26 (1.40)	2.58 (2.08)			7.92 (2.00)
		16	Girls	2632	1.18 (1.35)	1.93 (1.80)			8.50 (1.83)
TEDS	Self	16	Boys	2134	1.78 (1.52)	3.60 (2.32)	1.58 (1.46)	1.95 (1.86)	6.52 (1.97)
		16	Girls	2632	1.58 (1.44)	3.50 (2.28)	1.53 (1.46)	3.43 (3.32)	7.64 (1.77)

**Table 5:** Means and standard deviations for the scales of the Devereux Child Behaviour rating scale (DCB), Autism–Tics, ADHD and other Comorbidities inventory (A-TAC) and Multidimensional Peer Nomination Inventory (MPNI)

DCB	Rater	Age	Sex	N	Aggressive behaviour	Attention problems	Social isolation	Anxiety problems	Emotion liability	Dependency	Physical skill
NTR	Mother	5	Boy	7520	12.35 (3.77)	11.87 (3.57)	4.26 (1.45)	10.66 (3.29)	11.67 (3.50)	11.45 (3.05)	9.86 (3.13)
		5	Girl	7695	11.68 (3.42)	11.32 (3.44)	4.36 (1.40)	10.99 (3.40)	11.19 (3.33)	10.73 (2.87)	8.45 (2.71)
NTR	Father	5	Boy	6808	12.65 (3.84)	12.02 (3.34)	4.40 (1.47)	10.84 (3.22)	11.78 (3.29)	11.70 (3.03)	10.26 (3.13)
		5	Girl	6985	12.05 (3.55)	11.61 (3.26)	4.46 (1.43)	11.23 (3.30)	11.40 (3.22)	11.11 (2.85)	9.05 (2.88)

A-TAC	Rater	Age	Sex	N	CD	ADHD	Autism	ODD
CATSS	Parent	9	Boy	5610	0.11 (0.39)	2.50 (3.42)	1.00 (1.83)	0.52 (0.91)
		9	Girl	5516	0.08 (0.32)	1.65 (2.72)	0.63 (1.33)	0.41 (0.81)
CATSS	Parent	12	Boy	1649	0.11 (0.11)	2.39 (3.25)	0.99 (1.82)	0.49 (0.87)
		12	Girl	1598	0.05 (0.24)	1.36 (2.34)	0.60 (1.24)	0.32 (0.66)

MPNI	Rater	Age	Sex	N	Aggression	Inattention	Hyperactive–impulsivity	Social anxiety	Depression	Prosocial
FT12	Parent	12	Boy	1188	0.63 (0.42)	0.82 (0.52)	0.82 (0.54)	0.79 (0.61)	0.75 (0.43)	1.93 (0.37)
		12	Girl	1173	0.54 (0.39)	0.57 (0.45)	0.63 (0.47)	0.87 (0.61)	0.78 (0.43)	2.03 (0.37)

### Measures

The *Child Behaviour Checklist* (CBCL) 1,5–5 [26] and 6–18 [22] were used by GenR (age 6 and 10), TCHAD (ages 8, 13 and 16) and NTR (ages 3, 7, 9 and 12). The *Youth Self-Report* (YSR) [22] was used by TCHAD (ages 13 and 16). The CBCL and YSR are part of the Achenbach System of Empirical-Based Assessment and designed to measure childhood and adolescent behavioural and emotional problems. The response format was on a three-point scale (with response options ‘not true’, ‘somewhat true or sometimes true’ and ‘very or often true’). With the CBCL 1,5–5 seven syndrome scales are obtained (emotionally reactive, anxious–depressed, somatic complaints, withdrawn, overactive behaviour, aggressive behaviour, sleep problems), while with the CBCL 6–18 eight syndrome scales are obtained (anxious–depressed, withdrawn, somatic complaints, social problems, thought problems, attention problems, rule-breaking behaviour, aggressive behaviour). With the YSR, eight syndrome scales are obtained (anxious–depressed, somatic complaints, withdrawn–depressed, social problems, thought problems, attention problems, rule-breaking behaviour and aggressive behaviour).

The Strengths and Difficulties Questionnaire (SDQ) [23] was used by TEDs (ages 4, 7, 9, 16) and CATSS (age 15). The SDQ is a 25-item questionnaire designed to measure common mental health problems during childhood and adolescence. Ratings were on a three-point scale (with response options ‘not true’, ‘somewhat true’ and ‘certainly true’). The 25 items form 5 scales, emotional symptoms, conduct problems, hyperactivity/inattention, peer relationship problem and prosocial behaviour. The conduct problem scale was used as a proxy for aggressive behaviour.

NTR used the short *Devereux Child Behaviour (DCB) rating scale* for 5 year olds. The DCB consists of questions about problem behaviour in children rated by the parents [27]. The short version includes 42 items that measure seven different aspects of problem behaviour in children. Parents were asked to indicate on a five-point scale whether the statements were applicable (0 = never, 1 = rarely, 2 = occasionally, 3 = frequently, 4 = very frequently). The items of the questionnaire cover the following aspects of problem behaviour: emotional liability (five items, e.g. “markedly impatient”), social isolation (three items, e.g. “quite timid or shy”), aggressive behaviour (seven items, e.g. “hits, bites and scratches other children”), attention problems (five items, e.g. “jumps from one activity to another”), dependency (five items, e.g. “does not want to do things for himself”), anxiety problems (six items, e.g. “concern about his physical health”) and physical coordination (five items, e.g. “gets dirty and untidy”).

In 9- and 12-year-old in the CATSS sample from Sweden, information on ODD/CD and other psychopathologies was gathered through a telephone interview with parents, using *The Autism*–*Tics, ADHD and other Comorbidities inventory (A-TAC)* [24]. A-TAC is a comprehensive screening interview for autism spectrum disorders (ASDs), attention-deficit/hyperactivity disorder (AD/HD), tic disorders (TD), developmental coordination disorder (DCD), learning disorders (LD) and other childhood mental disorders that have been associated with these neurodevelopmental disorders.

In the FinnTwin12 sample from Finland, aggressive behaviour was assessed at ages 12, 14 and 17 by versions of the Multidimensional Peer Nomination Inventory (MPNI). The MPNI includes 37 items comprising three subscales, the two subscales used here include: externalising behavioural problems (aggression, hyperactivity–impulsivity and inattention) and internalising emotional problems (anxiety and depression) [28]. For each question (e.g. ‘Does the child tease smaller or weaker children?’), the informant rated how well the description fit the twin in question on a scale from 0 (the characteristic does not fit the child at all) to 3 (the characteristic fits the child very well). Parents rated the children at age 12, and the child rated him or herself at ages 14 and 17 years.

### Analyses

To ensure homogenous handling of data and identical analyses, all cohorts received a standard operating procedure that specified details of the comorbidity analyses. Following the SOP average scores and Pearson correlations for aggression with all other scales assessing psychopathology was obtained by a local analyst using their preferred statistical software. Average scores and correlations were computed by gender and age of children, separately for each rater and country. Results were uploaded to a shared server. Given the large datasets included in these analyses, leading to significance even if differences between average scores or between correlations being relatively small, we interpreted all results relative to each other and took the 95% confidence intervals into account. With the multi-instrument, multi-rater and multi-age assessments of aggression and of other emotional and behavioural problems, we established whether co-occurrence was stronger or weaker given different measurement instruments, raters and ages.

## Results

Tables 2, 3, 4 and 5 provide an overview of the sample sizes and mean levels of aggression and all other traits. We replicated the well-established gender differences in average aggression scores at most ages for parental ratings. The gender difference was smaller or close to absent for self-reports. For example, while the difference between boys and girls is in general about 1.5–2 points on the CBCL and SDQ parental reports, the differences based on self-report ranged between 0.05 and 0.67.

Mean levels based on similar instruments across countries were almost identical. For example, the mean level of aggression based on maternal ratings of 7-year-old boys in the Netherlands was 5.74 (SD 5.29), while mean level of aggression based on parental ratings of 8-year-old boys in Sweden was 5.49 (SD 5.42).

We observed differences between raters in nearly every country in the same direction. Based on maternal ratings, higher levels of psychopathology were seen than when based on paternal ratings. These differences were observed both for boys and girls, at ages 3–12 for the CBCL and SDQ. The exception was an absence of differences in maternal and paternal ratings when using the Devereux Child Behaviour rating scale.

With respect to our main question of the co-occurrence of aggression and other behavioural and emotional problems, findings are presented in Tables 6, 7, 8 and 9. Strong correlations were found between aggression and other externalising traits, especially rule-breaking behaviour. Correlations of almost similar strength were also observed for aggression and attention problems and hyperactivity. However, correlations were lower between aggression and internalising behaviours including withdrawn–depression and somatic complaints. Correlations between aggression and all other emotional and behavioural problems and their 95% confidence intervals are also provided in an interactive application which can be found at http://www.action-euproject.eu/ComorbidityChildAggression.

**Table 6:** Phenotypic correlations between aggression and other empirical scales of the Child Behaviour Checklist (CBCL) 1.5-5

ASBA-CBCL 1.5–5	Rater	Age	Sex	N	Attention problems	Withdrawn	Anxious– depressed	Emotional reactivity	Somatic complaints	Sleep problems
Gen R	Mother	3	Boys	2271	0.60 [0.57, 0.63]	0.43 [0.39, 0.47]	0.44 [0.40, 0.48]	0.67 [0.64, 0.70]	0.39 [0.35, 0.43]	0.38 [0.34, 0.42]
		3	Girls	2246	0.59 [0.56, 0.62]	0.42 [0.38, 0.46]	0.46 [0.42, 0.50]	0.68 [0.65, 0.71]	0.38 [0.34, 0.42]	0.40 [0.36, 0.44]
Gen R	Father	3	Boys	1840	0.67 [0.64, 0.70]	0.45 [0.41, 0.49]	0.47 [0.43, 0.51]	0.69 [0.66, 0.72]	0.39 [0.35, 0.43]	0.41 [0.37, 0.45]
		3	Girls	1897	0.59 [0.55, 0.63]	0.44 [0.40, 0.48]	0.50 [0.46, 0.54]	0.71 [0.68, 0.74]	0.39 [0.35, 0.43]	0.42 [0.38, 0.46]
NTR	Mother	3	Boys	9277	0.58 [0.56, 0.60]	0.45 [0.43, 0.47]	0.47 [0.45, 0.49]	0.64 [0.62, 0.66]	0.36 [0.34, 0.38]	0.35 [0.33, 0.37]
		3	Girls	9360	0.55 [0.53, 0.57]	0.41 [0.39, 0.43]	0.48 [0.46, 0.50]	0.65 [0.63, 0.67]	0.37 [0.35, 0.39]	0.38 [0.36, 0.40]
Gen R	Mother	6	Boys	2887	0.59 [0.56, 0.62]	0.58 [0.55, 0.61]	0.55 [0.52, 0.58]	0.73 [0.71, 0.75]	0.40 [0.37, 0.43]	0.41 [0.38, 0.44]
		6	Girls	2856	0.55 [0.52, 0.58]	0.50 [0.47, 0.53]	0.55 [0.52, 0.58]	0.75 [0.73, 0.77]	0.42 [0.39, 0.45]	0.41 [0.38, 0.44]

**Table 7:** Phenotypic correlations between aggression and other empirical scales of the achenbach system of empirically based assessment (ASEBA) 6–18

ASEBA 6–18	Rater	Age	Sex	N	Attention problems	Rule breaking	Social problems	Anxious– depressed	Withdrawn –depressed	Thought problems	Somatic complaints
NTR	Mother	7	Boys	5720	0.56 [0.54, 0.58]	0.69 [0.67, 0.71]	0.63 [0.61, 0.65]	0.48 [0.46, 0.50]	0.36 [0.34, 0.38]	0.47 [0.45, 0.49]	0.30 [0.28, 0.32]
		7	Girls	5853	0.53 [0.51, 0.55]	0.66 [0.64, 0.68]	0.63 [0.61, 0.65]	0.47 [0.45, 0.49]	0.36 [0.34, 0.38]	0.47 [0.45, 0.49]	0.32 [0.30, 0.34]
NTR	Father	7	Boys	4134	0.58 [0.56, 0.60]	0.66 [0.64, 0.68]	0.59 [0.57, 0.61]	0.46 [0.43, 0.49]	0.34 [0.31, 0.37]	0.44 [0.41, 0.47]	0.29 [0.26, 0.32]
		7	Girls	4182	0.55 [0.52, 0.58]	0.61 [0.59, 0.63]	0.63 [0.61, 0.65]	0.49 [0.46, 0.52]	0.40 [0.37, 0.43]	0.45 [0.42, 0.48]	0.32 [0.29, 0.35]
TCHAD	Parent	8	Boys	552	0.66 [0.60, 0.72]	0.72 [0.66, 0.78]	0.58 [0.51, 0.65]	0.55 [0.48, 0.62]	0.36 [0.28, 0.44]	0.38 [0.30, 0.46]	0.23 [0.15, 0.31]
		8	Girls	534	0.56 [0.49, 0.63]	0.64 [0.57, 0.71]	0.58 [0.51, 0.65]	0.50 [0.43, 0.57]	0.41 [0.33, 0.49]	0.36 [0.28, 0.44]	0.29 [0.21, 0.37]
NTR	Mother	9	Boys	4543	0.54 [0.52, 0.56]	0.70 [0.68, 0.72]	0.63 [0.61, 0.65]	0.49 [0.46, 0.52]	0.38 [0.35, 0.41]	0.44 [0.41, 0.47]	0.27 [0.24, 0.30]
		9	Girls	4689	0.54 [0.52, 0.56]	0.67 [0.65, 0.69]	0.66 [0.64, 0.68]	0.53 [0.51, 0.55]	0.43 [0.40, 0.46]	0.47 [0.44, 0.50]	0.31 [0.28, 0.34]
NTR	Father	9	Boys	3210	0.54 [0.51, 0.57]	0.70 [0.68, 0.72]	0.60 [0.57, 0.63]	0.49 [0.46, 0.52]	0.40 [0.37, 0.43]	0.48 [0.54, 0.51]	0.27 [0.24, 0.30]
		9	Girls	3255	0.53 [0.50, 0.56]	0.63 [0.60, 0.66]	0.63 [0.60, 0.66]	0.52 [0.49, 0.55]	0.43 [0.40, 0.46]	0.44 [0.41, 0.47]	0.30 [0.27, 0.33]
Gen R	Mother	10	Boys	2250	0.57 [0.54, 0.60]	0.72 [0.69, 0.75]	0.64 [0.61, 0.67]	0.52 [0.48, 0.56]	0.43 [0.39, 0.47]	0.56 [0.53, 0.59]	0.31 [0.27, 0.35]
		10	Girls	2310	0.53 [0.50, 0.56]	0.63 [0.60, 0.66]	0.64 [0.61, 0.67]	0.53 [0.50, 0.56]	0.42 [0.38, 0.46]	0.54 [0.51, 0.57]	0.33 [0.29, 0.37]
Gen R	Father	10	Boys	1624	0.60 [0.56, 0.64]	0.72 [0.69, 0.75]	0.64 [0.60, 0.68]	0.51 [0.47, 0.55]	0.47 [0.43, 0.51]	0.60 [0.56, 0.64]	0.28 [0.23, 0.33]
		10	Girls	1670	0.52 [0.48, 0.56]	0.62 [0.58, 0.66]	0.60 [0.56, 0.64]	0.54 [0.50, 0.58]	0.42 [0.38, 0.46]	0.57 [0.53, 0.61]	0.27 [0.22, 0.32]
NTR	Mother	12	Boys	3870	0.56 [0.53, 0.59]	0.71 [0.69, 0.73]	0.60 [0.57, 0.63]	0.51 [0.48, 0.54]	0.42 [0.39, 0.45]	0.46 [0.43, 0.49]	0.28 [0.25, 0.31]
		12	Girls	4010	0.54 [0.51, 0.57]	0.66 [0.64, 0.68]	0.62 [0.60, 0.64]	0.55 [0.52, 0.58]	0.43 [0.40, 0.46]	0.41 [0.38, 0.44]	0.33 [0.30, 0.36]
NTR	Father	12	Boys	2764	0.60 [0.57, 0.63]	0.73 [0.70, 0.76]	0.61 [0.58, 0.64]	0.49 [0.46, 0.52]	0.42 [0.39, 0.45]	0.48 [0.45, 0.51]	0.32 [0.28, 0.36]
		12	Girls	2839	0.55 [0.52, 0.58]	0.64 [0.61, 0.67]	0.63 [0.60, 0.66]	0.51 [0.48, 0.54]	0.43 [0.40, 0.46]	0.39 [0.36, 0.42]	0.30 [0.26, 0.34]
TCHAD	Parent	13	Boys	535	0.59 [0.52, 0.66]	0.60 [0.53, 0.67]	0.46 [0.38, 0.54]	0.51 [0.44, 0.58]	0.31 [0.23, 0.39]	0.22 [0.14, 0.30]	0.28 [0.20, 0.36]
		13	Girls	522	0.70 [0.64, 0.76]	0.68 [0.62, 0.74]	0.62 [0.55, 0.69]	0.67 [0.61, 0.73]	0.52 [0.45, 0.59]	0.39 [0.31, 0.47]	0.44 [0.36, 0.52]
TCHAD	Self	13	Boys	560	0.58 [0.51, 0.65]	0.58 [0.51, 0.65]	0.44 [0.37, 0.51]	0.46 [0.39, 0.53]	0.31 [0.23, 0.39]	0.31 [0.23, 0.39]	0.35 [0.27, 0.43]
		13	Girls	551	0.54 [0.47, 0.61]	0.52 [0.45, 0.59]	0.34 [0.26, 0.42]	0.43 [0.35, 0.51]	0.21 [0.13, 0.29]	0.38 [0.30, 0.46]	0.35 [0.27, 0.43]
TCHAD	Parent	16	Boys	532	0.58 [0.51, 0.65]	0.62 [0.55, 0.69]	0.41 [0.33, 0.49]	0.47 [0.39, 0.55]	0.30 [0.22, 0.38]	0.28 [0.20, 0.36]	0.36 [0.28, 0.44]
		16	Girls	507	0.67 [0.61, 0.73]	0.73 [0.67, 0.79]	0.43 [0.35, 0.51]	0.49 [0.41, 0.57]	0.43 [0.35, 0.51]	0.34 [0.26, 0.42]	0.34 [0.26, 0.42]
TCHAD	Self	16	Boys	583	0.56 [0.49, 0.63]	0.56 [0.49, 0.63]	0.40 [0.33, 0.47]	0.38 [0.30, 0.46]	0.24 [0.16, 0.32]	0.39 [0.32, 0.46]	0.29 [0.21, 0.37]
		16	Girls	606	0.56 [0.49, 0.63]	0.52 [0.45, 0.59]	0.36 [0.29, 0.43]	0.34 [0.27, 0.41]	0.22 [0.14, 0.30]	0.35 [0.28, 0.42]	0.34 [0.27, 0.41]

**Table 8:** Phenotypic correlations between aggression and other scales of the Strengths and Difficulties Questionnaire (SDQ)

SDQ	Rater	Age	Sex	N	Hyperactivity	Peer problems	Emotion–anxiety	Prosocial
TEDS	Parent	4	Boys	3581	0.43 [0.40, 0.46]	0.22 [0.19, 0.25]	0.24 [0.21, 0.27]	− 0.29 [− 0.32, − 0.26]
		4	Girls	3788	0.41 [0.38, 0.44]	0.21 [0.18, 0.24]	0.26 [0.23, 0.29]	− 0.30 [− 0.33, − 0.27]
TEDS	Parent	7	Boys	2740	0.44 [0.41, 0.47]	0.26 [0.22, 0.30]	0.24 [0.20, 0.28]	− 0.26 [− 0.30, − 0.22]
		7	Girls	2892	0.40 [0.37, 0.43]	0.23 [0.19, 0.27]	0.26 [0.22, 0.30]	− 0.26 [− 0.30, − 0.22]
TEDS	Parent	9	Boys	1055	0.44 [0.39, 0.49]	0.27 [0.21, 0.33]	0.33 [0.27, 0.39]	− 0.25 [− 0.31, − 0.19]
		9	Girls	1245	0.45 [0.40, 0.50]	0.31 [0.26, 0.36]	0.28 [0.23, 0.33]	− 0.27 [− 0.32, − 0.22]
TEDS	Self	9	Boys	1055	0.45 [0.40, 0.50]	0.26 [0.20, 0.32]	0.34 [0.28, 0.40]	− 0.27 [− 0.33, − 0.21]
		9	Girls	1245	0.43 [0.38, 0.48]	0.29 [0.24, 0.34]	0.37 [0.32, 0.42]	− 0.23 [− 0.28, − 0.18]
TEDS	Parent	12	Boys	1828	0.46 [0.42, 0.50]	0.28 [0.24, 0.32]	0.29 [0.25, 0.33]	− 0.29 [− 0.33, − 0.25]
		12	Girls	2117	0.44 [0.40, 0.48]	0.27 [0.23, 0.31]	0.29 [0.25, 0.33]	− 0.34 [− 0.38, − 0.30]
TEDS	Self	12	Boys	1828	0.53 [0.49, 0.57]	0.27 [0.23, 0.31]	0.28 [0.24, 0.32]	− 0.26 [− 0.30, − 0.22]
		12	Girls	2117	0.50 [0.46, 0.54]	0.29 [0.25, 0.33]	0.36 [0.32, 0.40]	− 0.24 [− 0.28, − 0.20]
CATSS	Parent	15	Boys	2083	0.52 [0.48, 0.56]	0.25 [0.21, 0.29]	0.29 [0.25, 0.33]	− 0.36 [− 0.40, − 0.32]
		15	Girls	2199	0.58 [0.48, 0.56]	0.28 [0.24, 0.32]	0.39 [0.35, 0.43]	− 0.45 [− 0.49, − 0.41]
CATSS	Self	15	Boys	2258	0.43 [0.39, 0.47]	0.21 [0.17, 0.25]	0.24 [0.20, 0.28]	− 0.24 [-0.28, − 0.20]
		15	Girls	2806	0.44 [0.41, 0.47]	0.19 [0.15, 0.23]	0.29 [0.28, 0.29]	− 0.31 [− 0.35, − 0.27]
TEDS	Parent	16	Boys	2134	0.54 [0.50, 0.58]			− 0.38 [− 0.42, − 0.34]
		16	Girls	2632	0.51 [0.48, 0.54]			− 0.42 [− 0.45, − 0.39]
TEDS	Self	16	Boys	2134	0.45 [0.41, 0.49]	0.18 [0.14, 0.22]	0.26 [0.22, 0.30]	− 0.22 [− 0.26, − 0.18]
		16	Girls	2632	0.46 [0.43, 0.49]	0.25 [0.21, 0.29]	0.27 [0.23, 0.31]	− 0.25 [− 0.29, − 0.21]

**Table 9:** Phenotypic correlations between aggression and other scales of the Devereux Child Behaviour rating scale (DCB), Autism–Tics, ADHD and other Comorbidities inventory (A-TAC) and multidimensional peer nomination inventory (MPNI)

DCB	Rater	Age	Sex	N	Attention problems	Social isolation	Anxiety problems	Emotional lability	Dependency	Physical coordination problems
NTR	Mother	5	Boys	7520	0.37 [0.35, 0.39]	0.14 [0.12, 0.16]	0.35 [0.33, 0.37]	0.52 [0.50, 0.54]	0.05 [0.03, 0.07]	0.30 [0.28, 0.32]
		5	Girls	7695	0.36 [0.34, 0.38]	0.07 [0.05, 0.09]	0.34 [0.32, 0.36]	0.52 [0.50, 0.54]	0.03 [0.01, 0.05]	0.29 [0.27, 0.31]
NTR	Father	5	Boys	6808	0.36 [0.34, 0.38]	0.15 [0.13, 0.17]	0.36 [0.34, 0.38]	0.50 [0.48, 0.52]	0.05 [0.03, 0.07]	0.33 [0.31, 0.35]
		5	Girls	6985	0.37 [0.35, 0.39]	0.09 [0.07, 0.11]	0.39 [0.37, 0.41]	0.53 [0.51, 0.55]	0.06 [0.04, 0.08]	0.32 [0.30, 0.34]

A-TAC	Rater	Age	Boys girls	N	ADHD	ODD	Autism	OCD
CATSS	Parent	9	Boys	5610	0.46 [0.44, 0.48]	0.51 [0.49, 0.43]	0.46 [0.44, 0.48]	0.19 [0.16, 0.22]
		9	Girls	5516	0.45 [0.43, 0.47]	0.49 [0.47, 0.51]	0.44 [0.42, 0.46]	0.17 [0.14, 0.20]
CATS	Parent	12	Boys	1649	0.38 [0.34, 0.42]	0.47 [0.43, 0.51]	0.38 [0.34, 0.42]	0.16 [0.11, 0.21]
		12	Girls	1598	0.37 [0.32, 0.42]	0.41 [0.37, 0.45]	0.32 [0.27, 0.37]	0.05 [0.00, 0.10]

MPNI	Rater	Age	Sex	N	Inattention	Hyperactive– impulsivity	Social anxiety	Depression	Prosocial
FT12	Parent	12	Boys	1188	0.38 [0.33, 0.43]	0.52 [0.47, 0.57]	0.14 [0.08, 0.20]	0.27 [0.22, 0.32]	− 0.34 [− 0.39, − 0.29]
		12	Girls	1173	0.41 [0.36, 0.46]	0.50 [0.45, 0.55]	0.14 [0.08, 0.20]	0.29 [0.24, 0.34]	− 0.30 [− 0.35, − 0.25]

Some more remarkable findings included the relatively low correlation between aggression and obsessive–compulsive behaviour and the similarly relatively low correlation between aggression and social isolation and aggression and dependency. We, furthermore, observed a relatively low correlation between aggression and peer problems from the SDQ (ranging from 0.18 to 31). However, CBCL social problems showed stronger correlations with aggression (ranging from 0.34 to 0.66).

The overarching picture that emerged suggests that correlations are largely stable across rater and age. If any change is observed, it is indicative of stronger correlations when children grow older. The correlations patterns of boys are markedly similar to the correlational patterns of girls. The only exception was the ATAC-based correlation between ODD/CD and OCD based on parental ratings at age 12. Correlations were stronger when based on the CBCL in comparison to the other measures, especially for parental ratings, while the ATAC, which is a clinical interview rather than a survey, provided somewhat lower correlations. The Devereux Child Behaviour (DCB) rating scale provides the interesting finding of similar strength in correlations between aggressive behaviour and attention problems and anxiety problems, but also with physical coordination problems.

## Discussion

One of the aims of ACTION is to describe and improve the understanding of the co-occurrence of aggression with other forms of childhood psychopathology. Here, we presented the correlations of aggression with other psychopathologies in large European samples of children between ages 3 and 16 years old. We showed that aggression co-occurred with almost all other behavioural and social problems. More specifically, aggression co-occurred with oppositional and ADHD-related problems, and at later ages with rule-breaking. In addition to the high correlations of aggression with externalising problems, we also observed substantial associations with anxiety–depression and other internalising symptoms. This co-occurrence of internalising and externalising problems has previously been shown to persist over childhood and adolescence [29]. Both for externalising and internalising problems, the patterns of co-occurrence were largely gender and rater independent, and were similar even when aggression and the other psychopathologies were assessed by different instruments, such as the CBCL and the SDQ. Also, there were large similarities in co-occurrence patterns across countries in the Northern part of Europe.

In ACTION, we compared co-occurrence patterns across different countries and cultures. These comparisons are somewhat hampered by the fact that in almost all cases more than one parameter varies between the different countries and cultures. For example, both NTR, a Dutch sample, and TEDs, a UK sample, have parental ratings at age 9, but NTR used the CBCL while TEDS used the SDQ. Any differences in correlations may thus be attributable to cultural differences or country differences between the Netherlands and the UK, instrument differences or any other protocol or unobserved difference. However, given all these sources of difference in this large co-occurrence study, it is even more striking that most correlations are so similar.

The large associations of aggression with other emotional and behavioural problems may form one of the obstacles for prevention and treatment of aggression. These findings indicate that an exclusive focus on aggression might not be the most feasible approach for the development of effective prevention and intervention programs. The complexity of psychopathology, partly due to the co-occurrence of behavioural and emotional problems, needs to be addressed and its aetiology explored through genetic, longitudinal and causal modelling: do the strong associations of aggression and other emotional and behavioural problems reflect a shared genetic vulnerability for multiple disorders, or do some disorders causally lead to other problems?

The absence of rater differences in co-occurrence patterns does not imply that rater’s views are interchangeable. Previous research suggested that, in general, mothers observe more behaviour problems in their children than fathers do [30]. We also see this pattern in the current paper, and consistently observe it across all counties. The differences in assessment between fathers and mothers in the levels of behavioural problems they observe may indicate that they both introduce their rater-specific view on the behaviour of the child [31], or that fathers and mothers interact with their offspring in different contexts.

The similarities across raters and countries indicate that large-scale gene-finding efforts of aggressive behaviour and its co-occurring psychopathologies across multiple cohorts will be feasible/successful. Such an effort is currently in progress within the ACTION consortium in collaboration with other cohorts and consortia that have collected measures of aggression in children as well as DNA samples for genotyping [32]. The results of this international genome-wide association meta-analysis (GWAMA) are expected to yield insight into the genetic variants that influence aggression across childhood and offer possibilities for the construction of polygenic scores which may be used in prediction models [33, 34] and gene-environment modelling [35]. Besides a GWAMA approach, which includes samples from multiple age groups, genome-wide epigenetic profiling will be done to compare methylation in several statistically well-powered contrasts (such as genetically identical twin pairs discordant for aggression) in children. Monozygotic (MZ) twins pairs who are longitudinally discordant of aggression, also offer a unique possibility to gain an understanding of the environmental risk factors associated with complex behaviour such as aggression [36].

Genetic and epigenetic effects do not act in isolation, so the results of these studies will need to be investigated in (epi)gene x environmental interplay models to understand the differences between children in aggression. Twin data may offer a first insight into the importance of gene-environment dependencies. Analyses of behavioural problems in 5-year-old twins showed strong evidence for larger environmental influences in children who were genetically more at risk for problem behaviour [37]. The available large-scale phenotypic, environmental and genotypic databases in ACTION will allow the development and application of these methods for gene-environment interaction and correlation.

Although it is known that co-occurrence is a risk factor for persisting symptoms (e.g. [38]), the implications for treatment are under-investigated. The current paper underlines that co-occurrence of behavioural and emotional problems with childhood aggression is highly prevalent. Instead of excluding children with multiple problems, specific trials should be undertaken to investigate the effectiveness of treatment and improve treatment for this group that requires our utmost attention. Of course, the question then arises what would be more effective, e.g. treatment targeting all psychopathologies at the same time or treatment at symptom level. It is essential to gain knowledge about the etiological and sequential effects of the comorbid disorders. If one disorder also is found to precede another disorder, treatment can be adjusted and specified. To be able to initiate such treatment specificity, we need to conduct cross-lag longitudinal analyses to examine whether aggression is driving the other psychopathologies, or if aggression is a result or outing of other problems. If one set of symptoms drives the rest, then intervention should focus on early detection and prevention.

We conclude that childhood aggression co-occurs with nearly all other behavioural, emotional and social problems, from both externalising and internalising domains, regardless of rater, gender, measurement instrument or country. These findings indicate that aggression during childhood and adolescence rarely occur in isolation, and that other behavioural and emotional problems are common in children with aggressive problems.

## Future progress

**Fig. 1:**
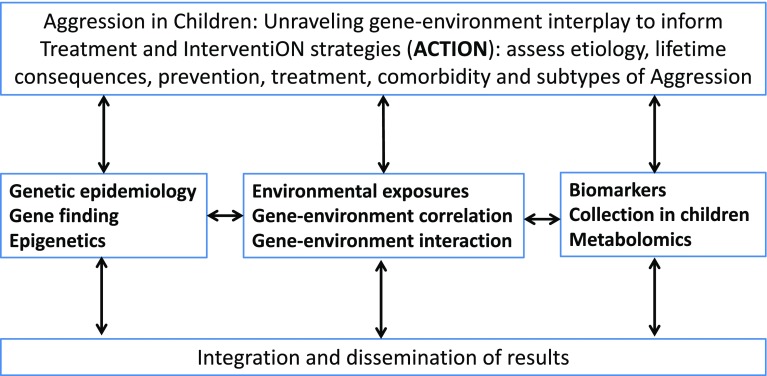
Work plan strategy of ACTION

The finding that aggression co-occurs with nearly all other behavioural, emotional and social problems during childhood puts aggression in the centre of scientific attention. If and when causes of differences in aggression during childhood are better understood, this information may aid in the development of prevention and intervention strategies. To this end, we designed the EU-ACTION project (see Fig. 1). The main objective of ACTION is to improve the understanding of the causes of individual differences in aggression among children to better inform the development of prevention and treatment strategies.

ACTION has described current clinical practices in Europe with respect to childhood aggression and identified drawbacks in prevention and intervention of clinical aggression (also known as paediatric conduct disorders). An online semi-structured questionnaire investigating the status of national guidelines (*N* = 29 academic experts; 23 countries) and an online semi-structured questionnaire exploring clinical practices (*N* = 94 clinicians; 22 countries) on diagnosing and treating children with severe behavioural problems across Europe were developed. Several countries have official clinical guidelines, while others have at least some unofficial documents. In general, primary and secondary preventions were absent or poorly developed, whereas specific interventions for severe behavioural problems were very diverse across Europe. Improving parent–child interactions, parent/teacher interventions and collaborative approaches were most frequently identified as successful treatment elements. Several needs were listed by experts and clinicians, which will fuel further research within ACTION and beyond. The current findings on co-occurrence of aggression indicates that information on these current drawbacks could also be informative for other psychopathologies.

A challenge in combining large cohort studies carried out in different countries is the assessment of aggression. Within different countries and cohorts, different instruments are used. In a subsample of the Netherlands Twin Register, we have invited a group of parents of 9-year-old twins to complete multiple assessment instruments to have a ‘reference set’ or ‘backbone’ for phenotype imputation.

The first results with respect to genetic and environmental contributions to the variation and longitudinal stability in childhood aggressive behaviour [39] indicated high stability and heritability of aggressive behavioural problems. Heritability was on average around 60–80% without any large gender differences in the magnitude of genetic effects. In boys, shared environment explained around 20% of the variation in aggression across all ages, while in girls its influence was absent around age 7 and only came into play at later ages. Longitudinal genetic correlations explained most of the stability of aggressive behaviour. These results are encouraging for gene-finding studies. In earlier work, the first molecular genetic evidence for aggression in childhood was reported [40]. Using genomic relationship matrix restricted maximum likelihood (GREML) analyses significant influences of common SNPs were estimated for externalising problems (SNP *h*^2^ = 0.44), for attention problems (SNP *h*^2^ = 0.37–0.71) and total problems (SNP *h*^2^ = 0.18). A previous attempt to discover genomic locations of interest for childhood and adolescent aggression (*N* = 18,988) identified one region in chromosome 2 (2p12) at near genome-wide significance (top SNP rs11126630, *P* = 5.30 × 10^− 8^). The gene-based analysis indicated association with variation within AVPR1A with aggressive behaviour. It was concluded that common variants at 2p12 show suggestive evidence for association with childhood aggression [41]. To replicate this finding and to initiate new findings we will use newly developed multivariate genome-wide meta-analysis methodology, in which the power of sample overlap (e.g. due to having a paternal and maternal rating of the same child at the same age) is leveraged instead of omitted [42]. In line with the results, we include ADHD and ADHD-related problems, as well aggressive behaviour in this collaborative project. With this approach, we will be able to identify not only genomic regions of interest for aggression or ADHD, but also genomic regions that play a role in the co-occurrence of these psychopathologies.

In addition to existing genotype datasets, new DNA samples are collected for epigenetic research in clinical cases (children that are referred to child psychiatric clinics in the Netherlands) and in identical twins concordant and discordant for aggression. While DNA collection and epigenetic profiling in these children is in progress, we gained initial insight into the association between aggression and DNA methylation patterns by analysing available data on aggression available for adults [43]. DNA methylation was measured in whole blood by the Illumina HM450k array in more than 2000 adults for whom Adult Self-Report [44] data on aggression were available. No genome-wide significant methylation hits were identified, but gene-ontology (GO) analysis, in which categories of genes rather than single methylation sites were tested, highlighted that genes involved in developmental and central nervous system processes are enriched among the higher-ranking genes from the epigenome-wide meta-analysis (EWAS). This study is now followed by a meta-analysis EWAS (EWAMA) in children and adults across multiple cohorts. This EWAMA includes multiple cohorts with a sample size of over 10.000.

In addition to genetic and environmental factors acting additively to the development of childhood aggression, genes and environment may interact. Such interactions can be thought of as genes controlling sensitivity to the environment, or as the environment controlling the expression of genes. Genes and environment may also be correlated when genes alter the exposure to relevant environmental risk factors. We know that for traits such as aggression children are not randomly distributed over environments and describing environmental effects as “causal” may lead to wrong conclusions/interventions. Several mechanisms can be at play to explain the non-random distribution of genotypes over environments [45]: children who inherit genes that make them susceptible to exhibiting aggression are likely to grow up in aggressive homes (passive rGE), their genotypes may trigger aggression in others (reactive rGE) and they may seek out aggressive peer groups (active rGE). The analyses of rGE thus are closely related to issues of gene-environment independence and to questions of causality. The analyses of GxE interaction will employ several approaches that can make use of the large existing datasets. The first approach focuses on the estimation of the total contribution of genes when environmental exposures have been measured. In this approach genotypes and other, non-measured, influences are modelled as latent factors. Because of the presence of genome-wide marker data, a second approach is to estimate GxE interaction in a design with measured genotypes and environmental exposures (note that because of the twin design the remaining variance can still be attributed to latent G and E). The causal relation of environmental exposure and later outcome may be complex, but longitudinal twin data offer excellent opportunities to test models of causality versus other models of association between genes and environment [46, 47].

A final piece of the puzzle is sought in the assessment if biomarker and metabolomics profiles in clinical cases and MZ twins discordant for aggression. Given the fact that human aggressive behaviour is heterogeneous and that most effective therapeutic agents only work on the serotonergic system a comprehensive study of the role of the amino acid neurotransmitters (including both their precursors and degradation metabolites) and peptide-based neurotransmitters is warranted. In addition to this biomarker approach, ACTION will include a metabolomics approach and the platforms we are measuring include amines, organic acids and steroids.

Results from ACTION will be integrated into an empirical-based framework of aggression. The sample sizes of ACTION will allow us to examine the interplay between risk factors and test hypotheses to identify modifiable risk factors for childhood aggression. Thereby, our findings may inform prevention and treatment strategies, and assist in individual risk profiles based on combination of modifiable and non-modifiable risk indicators. Translation of results will be supported by several internet applications and disseminating the results via the ACTION website (http://www.action-euproject.eu/).

## Appendix 1

### Participating cohorts

The Netherlands Twin Register (NTR) was established in 1987 and collects data in twins and multiples from birth onwards. Nationwide data collection is by mailed surveys to the parents of twins until age 12, and to twins after age 14. At age 14, siblings of twins are also invited to take part and at age 18 twins and their siblings and parent are invited to complete a series of self-report questionnaires. Parents of twins receive questionnaires when their twins are aged 1, 2, 3, 5, 7, 10 and 12 years of age. After 25 years of research, large datasets have been obtained. Information on behavioural and emotional problems throughout childhood is obtained by assessment with the Child Behaviour Checklist (CBCL), Teacher Report Form (TRF), Devereux Child Behaviour (DCB) rating scale and Youth Self-Report (YSR). This longitudinal data collection strategy has the advantage that multiple informant assessment can be easily combined, due to overlapping items by gender, informant and age. For each age group, items can be summed to form longitudinal syndrome scales and a total problem score. When twins reach age 18, they and their parents and siblings are invited to take part in the data collection as adult twin families. They receive an extensive survey, that includes the Adult Self-Report (ASR).

The Twins Early Development Study (TEDS) was established in 1995 with three birth cohorts (1994–1996) obtained from UK birth records. In infancy and early childhood, questionnaires were posted to parents and teachers (with permission from parents), and school achievement records were also obtained. Data were also obtained from telephone interviews and increasingly from online internet assessment. The measure used consistently at all ages and from all sources (including the twins themselves beginning at age 10) is the Strength and Difficulties Questionnaire (SDQ). The SDQ is particularly useful for combining information across informants and across ages. At various ages, we have assessed using a battery of measures other aggression-relevant domains, most notably psychopathic symptoms and attention-deficit/hyperactivity disorder symptoms. We are currently collecting only minimal information at age 18 and plan a major follow-up at age 21, which will serve our ACTION collaboration.

The Swedish Twin Register (STR) was established in 1961 and includes all 200,000 + twins born in Sweden since 1886. In the Swedish Twin study of Child and Adolescent Development (TCHAD), we have followed 1,500 twin pairs from age 8 to age 20 with 4 waves of questionnaires to both parents and twins (1994, 1999, 2002, 2006) and we just completed a follow-up (November 2013) at age 26. Information on behavioural and emotional problems throughout childhood is obtained by assessment with the Child Behaviour Checklist (CBCL), Teacher Report Form (TRF) and Youth Self-Report (YRF), together with more in-depth assessments of for example aggression (Youth Psychopathic traits Inventory, YPI; aggressive behaviour in youth). In the ongoing Child and Adolescent Twin Study in Sweden (CATSS) study, initiated in 2004, we conduct a psychiatric telephone interview with parents of all 1,400 twin pairs born in Sweden annually in connection with their 9th birthdays. By May 2016, we have performed 28,168 interviews with a very high response rate (more than 76%), and we have collected DNA from the twins (current *N* ≈ 14,500 individuals). We follow these families with questionnaires to parents and twins at age 15 (CATSS-15; current *N* = 11,148) and 18 (CATSS-18; current *N* = 7143 twins). Information on behavioural and emotional problems at age 9 is gathered through a telephone interview with parents using the A-TAC instrument which among others include ODD/CD modules. Aggression and criminality are measured through questionnaires to both parents and twins at age 15 and 18.

The FinnTwin12 study was started in September 1994 to examine genetic and environmental determinants of precursors to health-related behaviours, with a particular focus on the use and abuse of alcohol, in initially 11- to 12-year-old twins. This research is cast within the perspective of developmental genetic epidemiology, asking whether precursors of risk behaviours are evident to parents, teachers and classroom peers as early as age 12. Information on behavioural and emotional problems throughout childhood is obtained from in-person psychiatric interviews using the Semi-Structured Assessment for the Genetics of Alcoholism (SSAGA) and by questionnaire assessment with the Multidimensional Peer Nomination Inventory (MPNI).​ The MPNI was designed by Finnish psychologist Dr Lea Pulkkinen, arising from work started in 1968, and evolved into the 37 item questionnaire used in the first three waves of assessment. The MPNI gathers information on three major dimensions: Behavioural Problems (aggression [both direct and indirect], hyperactivity–impulsivity and inattention), Emotional Problems (depression, social anxiety and victimisation) and Adjustment (constructiveness, compliance, helping behaviour and social activity). The study has a two-stage sampling design. The larger, first-stage study is an epidemiological investigation of five consecutive and complete birth cohorts (1983–1987) of Finnish twins, including questionnaire assessments of both twins and parents at baseline, starting with a family questionnaire (returned by 2,724 families, 87% participation rate) that was mailed late in the year before the twins reached age 12, with follow-up of all twins at age 14 and 17½, as well as a later collection of questionnaires, psychiatric interviews and blood samples at age 22. For the epidemiological study of the first wave of data collection, we excluded families in which one or both co-twins were deceased or living outside Finland, families in which both co-twins lived apart from both biological parents, and families in which the Population Register Center contained no residential address for a twin.

The Generation R Study from Rotterdam in the Netherlands is a population-based prospective cohort study from foetal life until young adulthood. The study is designed to identify early environmental and genetic causes of normal and abnormal growth, development and health during foetal life, childhood and adulthood. The study focuses on four primary areas of research: (1) growth and physical development; (2) behavioural and cognitive development; (3) diseases in childhood; and (4) health and healthcare for pregnant women and children. In total, 9,778 mothers with a delivery date from April 2002 until January 2006 were enrolled in the study. General follow-up rates until the age of 4 years exceed 75%. Data collection in mothers, fathers and preschool children included questionnaires, detailed physical and ultrasound examinations, behavioural observations and biological samples. A genome-wide association screen is available in the participating children. Regular detailed hands-on assessments are performed from the age of 5 years onwards.

The Queensland Twin Register (Qtwin) study began in 1992 and collects data from twin and their siblings. Twins were recruited from primary and secondary schools in south east Queensland in Australia. Longitudinal data are collected from the twins, their siblings and their parents during visits to the Queensland Institue of Medical Research (QIMR Berghofer Medical Research Institute), which are scheduled as close as possible to the twins 12th, 14th and 16th birthdays. Data collection in the 21–22 year old studies is via online questionnaire and for a subset of individuals a semi-structured telephone interview. In addition, a number of focus studies have been conducted including the MRI study in which brain MRI and fMRI data were collected on ~ 1,200 individuals. Qtwin has also conducted a number of cross-sectional online questionnaire studies collecting data from the twins, and their siblings and from the twins’ mothers. Information on behavioural and emotional problems throughout childhood is obtained by assessment with the Swan and structured interviews and questionnaires based on the CIDI. The result is a rich and diverse longitudinal database which includes in-depth psycho-social, biological and environmental data.
